# A new layer of complexity in the human genome: Somatic recombination of repeat elements

**DOI:** 10.1002/ctm2.1226

**Published:** 2023-03-20

**Authors:** Giovanni Pascarella, Martin Frith, Piero Carninci

**Affiliations:** ^1^ RIKEN Center for Integrative Medical Sciences (IMS) Yokohama Japan; ^2^ Artificial Intelligence Research Center National Institute of Advanced Industrial Science and Technology (AIST) Tokyo Japan; ^3^ Graduate School of Frontier Sciences University of Tokyo Chiba Japan; ^4^ Computational Bio Big‐Data Open Innovation Laboratory (CBBD‐OIL) AIST Tokyo Japan; ^5^ Human Technopole Milan Italy

1

The establishment of sequencing technologies as major discovery tools in modern genomics has progressively expanded our knowledge of a myriad of DNA sequence variants existing within a single individual, ranging from one single base to megabases, and even entire chromosomes. These variations can emerge in our organisms at various stages of postzygotic development and throughout our existence, as they are mechanistically linked to ubiquitous pathways such as DNA repair and replication. The DNA contained within the nucleus is consistently exposed to both internal and external sources of damage, which can compromise its structural integrity. If left unaddressed, such DNA damage can trigger the activation of apoptotic pathways, ultimately leading to cell death. To avoid this undesirable outcome, all living organisms have evolved repair mechanisms aimed at healing DNA lesions and buffering their potential threat. The localized rupture of both DNA strand (double strand break, DSB) is one of the most common and severe forms of DNA damage, estimated to happen 10−50 times per day in each cell.^1^ A major pathway of DNA DSBs repair is homologous recombination (HR), that exploits the intact DNA sequence of one chromatid to repair the damaged locus on its sister chromatid.^2^ The search for the intact homologous DNA locus is a key step in HR, and it is controlled by several proteins specific to this repair pathway. However, eukaryotic genomes are composed of a wide array of repeat elements that may mislead the search for homologous loci and act as alternative templates for the repair, thus causing non‐allelic homologous recombination (NAHR).^3^ Depending on the genomic location of the sequences engaged in this process, NAHR can result in genomic inversions, deletions and duplications with the potential to disrupt the structure and function of the affected loci^4^; it is therefore not surprising that the bulk of NAHR characterized so far in the human genome is within the context of cancers and genetic disorders. Frequently, the breakpoints of such pathogenic NAHR events have been found in or around Alu and L1 repeats, that together account for ∼30% of the human genome sequence.^5^, ^6^, ^7^, ^8^ While in normal genomes NAHR has been considered for long as a rare occurrence, given the millions of Alu and L1 copies and the high frequency of DNA DSBs instances in the genome of each cell, we postulated that the number of NAHR events in human genomes might have been exceedingly underestimated.

To address this question, we combined a protocol^9^ for the enrichment of retrotransposons sequences (capture‐seq) with a novel bioinformatic tool (TE‐reX) to comprehensively investigate somatic NAHR events generated by Alu and L1 elements in post‐mortem tissues of different embryonic derivation, including liver, kidney, and cortical brain regions from ten neurotypical donors (Figure 1). Our approach allowed us to annotate more than 2.5 million somatic NAHR events across this dataset, thus demonstrating that the NAHR of Alu and L1 sequences is a frequent event in normal genomes. A thorough genome‐wide annotation of somatic NAHR events revealed a higher count of recombination in kidney and liver samples (∼4–5 estimated NAHR events per cell) compared to brain samples (∼1 NAHR per cell), but the tissue‐specific differences extended much further (Figure 2A). In fact, we discovered a striking enrichment of intra‐chromosomal NAHR in the brain compared to non‐brain samples; moreover, within each chromosome we found that the recombination of proximal repeats is enriched in the brain, suggesting different dynamics of NAHR compared to kidney and liver. We further observed that the NAHR of proximal repeats in inverted configuration (head‐to‐head or tail‐to‐tail) is more frequent than that of proximal repeats in direct orientation, and again this bias was detected as more prominent specifically in brain samples. The vast number of NAHR events discovered with our approach led us to look for NAHR hotspots genome‐wide; we thus found that several pericentromeric loci have a very high NAHR rate compared to surrounding loci. We also observed that “hot” Alu elements with high recombination activity are enriched in oncogenes and tumour suppressor genes, suggesting that in healthy cells NAHR may prime the genome toward disease development.

**FIGURE 1:**
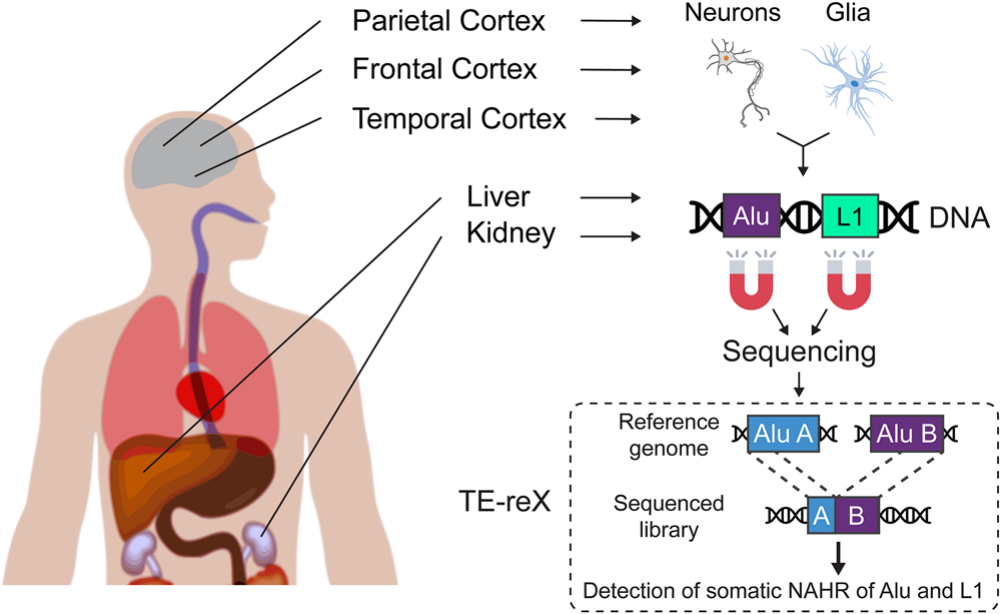
Experimental approach for the discovery of somatic non‐allelic homologous recombination (NAHR) of Alu and L1 repeat elements. Genomic DNA was purified from post‐mortem tissues of 10 neurotypical donors; for all brain samples, the purification was performed on neuronal and glial fractions isolated by nuclear sorting. Alu and L1 sequences were enriched using RNA probes, and the data for the sequenced capture‐seq libraries were used as input for the bioinformatic pipeline TE‐reX that discovers and annotates NAHR events involving Alu and L1.

**FIGURE 2:**
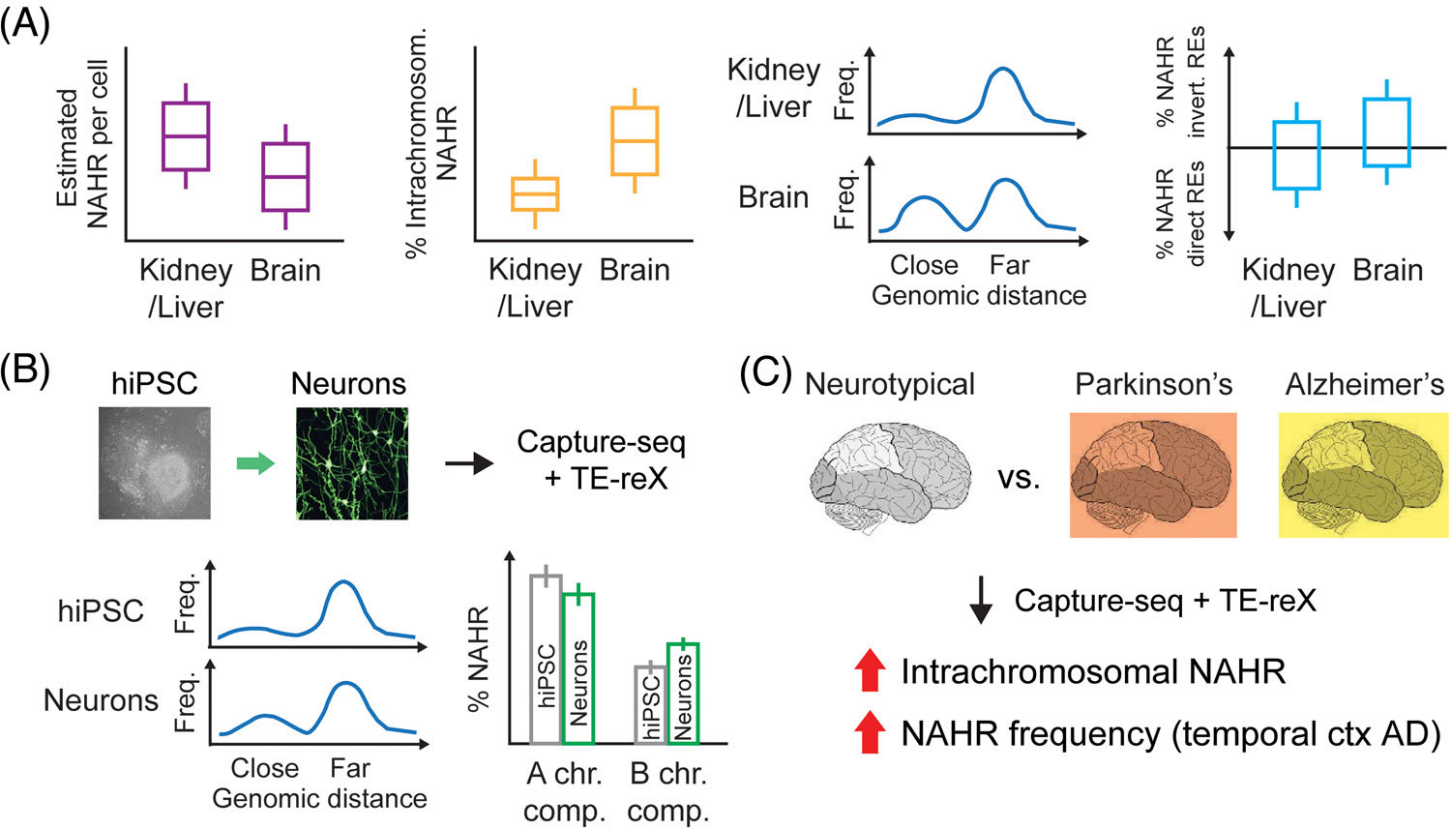
(A) Characteristics of tissue‐specific somatic non‐allelic homologous recombination (NAHR), detected by TE‐reX in capture‐seq libraries of neurotypical donors. Left to right: non‐brain samples have a higher estimated count of NAHR per cell; brain samples are enriched for intrachromosomal NAHR; brain samples show an enrichment of NAHR of proximal repeat elements; NAHR of proximal repeat elements in inverted orientation is more prominent in brain samples. **(B**) In vitro differentiation of hiPSC to neurons is accompanied by neuron‐specific changes of NAHR profiles, including an enrichment of NAHR of proximal repeats (bottom left), a depletion of NAHR in active chromatin compartments (“A”) and a concurrent enrichment of NAHR in inactive chromatin compartments (“B”) (bottom right). **(C**) Characterization of somatic NAHR in sporadic Parkinson's and Alzheimer's disease revealed tissue‐ and disease‐specific alterations of NAHR profiles compared to NAHR in neurotypical donors.

To understand whether the tissue‐specific NAHR profiles might be defined during development, we employed a model of human induced pluripotent stem cells (hiPSC) differentiated in neurons.^10^ By applying our NAHR discovery workflow to this in vitro system, we showed that the neuronal differentiation was accompanied by the emergence of neuron‐specific NAHR profiles, thus demonstrating that tissue‐specific recombination may be active during early developmental stages.

The recombination patterns detected in samples from neurotypical donors further prompted us to wonder if and how NAHR of Au and L1 might be altered in neurodegenerative disorders like Parkinson's (PD) and Alzheimer's disease (AD), where increased oxidative stress has been suggested to cause DNA damage and genomic instability.^11^ We repeated our analyses of NAHR of Alu and L1 in kidney, liver and brain samples from donors affected by sporadic cases of PD and AD; when compared to neurotypical donors, we found that PD and AD samples were characterized by disease‐specific and tissue‐specific alterations of somatic NAHR profiles, indicating a possible contribution of somatic NAHR of Alu and L1 to the pathogenesis and/or progression of neurodegenerative processes. Finally, we showed that somatic NAHR can be detected whole‐genome sequencing libraries sequenced on an Oxford Nanopore Technologies platform that does not require capture or library amplification by PCR. This important confirmation of NAHR demonstrated also that the bulk of NAHR events in the human genome is caused by Alu and L1 repeats, while other genomic repeats contribute to a very small extent.

A key question that stems from our findings is how much more NAHR may happen in our genomes on a shorter time scale; in fact, NAHR recovered from post‐mortem tissues are likely to be neutral for the host cell fitness, while other NAHR events with a worse outcome for the structure and function of the host genome may be subject to purifying selection, thus being absent from post‐mortem tissues. Our study expands the knowledge of the complexity of the human genome and shows that the DNA in our cells is remarkably plastic, being able to tolerate such a high rate of genomic recombination.

## CONFLICT OF INTEREST STATEMENT

The authors declare no competing interests.
